# Subacute Combined Degeneration Masking Spinal Stenosis: A Case Report

**DOI:** 10.7759/cureus.36367

**Published:** 2023-03-19

**Authors:** Palak R Patel, Randel L Swanson, Franklin E Caldera

**Affiliations:** 1 Physical Medicine and Rehabilitation, University of Pennsylvania Perelman School of Medicine, Philadelphia, USA; 2 Center for Neurotrauma, Neurodegeneration and Restoration, Corporal Michael J. Crescenz VA Medical Center, Philadelphia, USA

**Keywords:** gait ataxia, cervical myelopathy, vitamin b12 deficiency, progressive myelopathy, hickam’s dictum, occam's razor, cervical spinal stenosis, subacute combined degeneration

## Abstract

Subacute combined degeneration (SCD) from vitamin B12 deficiency and spinal stenosis from degenerative changes may present similarly with weakness, sensory disturbances, and ataxia but require different treatments. This case report describes a 74-year-old male with suspected SCD who was discharged to an inpatient rehabilitation facility (IRF), did not improve with B12 supplementation, and later developed signs of myelopathy and diffuse joint pain. He ultimately was found to have severe cervical stenosis and pseudogout that were treated with a laminectomy and colchicine, respectively. Following surgical intervention, he returned to the IRF, where he had considerable functional improvement and was safely discharged home. This report shows the importance of recognizing the two conditions, their overlap, and the contrast between Occam’s razor and Hickam’s dictum.

## Introduction

Subacute combined degeneration (SCD) from vitamin B12 deficiency presents with weakness, sensory disturbances, ataxia, and potentially frequent falls due to demyelination of the dorsal and lateral columns of the spinal cord [1]. Vitamin B12 is involved in DNA synthesis and the creation and maintenance of myelin present in the spinal cord. Deficiency can be seen in those who are vegan or vegetarian while not consuming regular B12 supplements or can be found in those with pernicious anemia. Workup is notable for macrocytic anemia, elevated methylmalonic acid (MMA) and homocysteine levels, and magnetic resonance imaging (MRI) with demyelination in the dorsal and lateral columns. The condition is usually reversible with B12 supplementation, with 86% showing clinical resolution within a year [1].

Spinal stenosis, commonly from degenerative changes, also presents with weakness, pain, and sensory changes. Specifically, in patients with greater than 30% cervical stenosis, spondylotic myelopathy may be present with upper extremity weakness, difficulties in ambulation, and ataxia [2]. This case describes the overlap of these conditions and illustrates the consequence of not recognizing their concurrent presence.

## Case presentation

The patient is a 74-year-old male with a history of diabetes, hypertension, and hyperlipidemia who presented to the emergency department with worsening balance and frequent falls. One year prior to this, the patient presented to another hospital with similar symptoms and was found to have a vitamin B12 deficiency, with labs significant for hemoglobin of 12.2 g/dL with mean corpuscular volume (MCV) of 83.4 fl, vitamin B12 level of 107 pg/mL (low), MMA of 351 nmol/L (high), and homocysteine of 13.5 µmol/L (high). Cervical x-ray at this time showed multilevel degenerative changes, including moderate to severe neuronal foraminal stenosis, but he was unable to undergo MRI due to retained bullet fragments from a previous gunshot wound. Neurology was consulted and felt the primary etiology for the clinical presentation was SCD, and further imaging was not pursued. The patient was instructed to return to his primary care provider for B12 injections but was lost to follow-up.

On this current presentation, he was admitted to the neurology department, with an exam significant for bilateral hand paresthesias, weakness in bilateral lower extremities with increased tone and decreased proprioception, and labs significant for hemoglobin of 10.2 g/dL with MCV of 75.4 fl, vitamin B12 level of 177 pg/mL (low), MMA of 172 nmol/L (normal), without evidence for intrinsic factor blocking antibody, ruling out pernicious anemia. He was treated with cyanocobalamin intramuscular injections, followed by oral supplementation. He also became febrile during the admission and tested positive for COVID-19 but was otherwise asymptomatic from a respiratory standpoint. 

He was later admitted to an inpatient rehabilitation facility (IRF) to help regain his strength and balance. At initial evaluation, he had full strength in all extremities, some dysmetria with numbness, and could ambulate with a rolling walker. The following day, he developed severe right ankle pain, which was swollen and tender to palpation, with increased difficulty in ambulation. The next day, he then developed severe left ankle pain. He also became very lethargic and was found to be febrile to 102.6°F. Infectious disease was consulted, and a full workup was unremarkable, so antibiotics were deferred. Over the course of his 11-day admission, he became progressively weaker and could not tolerate physical and occupational therapy. Therapists noted a gross decline in overall function, where he previously required minimal assistance (25% external effort needed) for functional transfers and short-distance ambulation and regressed to maximal assistance (75%) to dependent (100%) for transfers requiring an overhead lift. He then also developed left wrist pain with swelling and tenderness. Blood work was pertinent for increased inflammation with a sedimentation rate of 129 mm/H, c-reactive protein of 156 mg/L, platelets of 415 tho/uL, aspartate aminotransferase of 81 U/L, and alanine transaminase of 93 U/L. Neurology was consulted at the IRF and felt the patient required immediate transfer to acute care, including admission to neurology for a CT myelogram, CSF studies, and autoimmune workup. 

**Figure 1:**
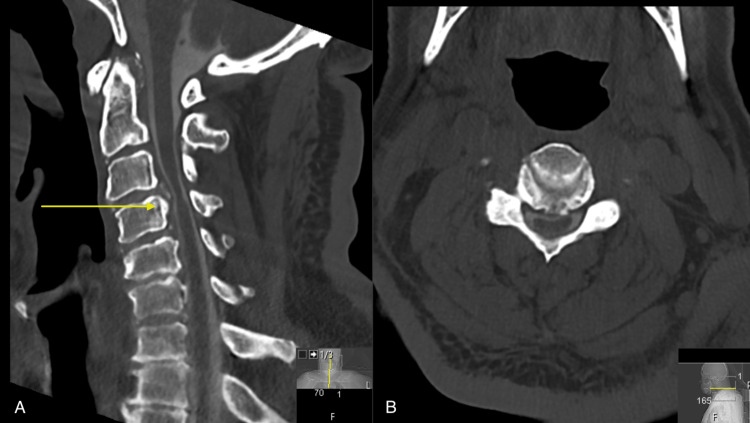
CT Cervical Spine with Myelographic Contrast (A) Single sagittal slice of a CT of the cervical spine with myelographic contrast demonstrating severe spinal canal stenosis secondary to a large disc osteophyte complex which indents the ventral thecal sac and results in compression of the ventral aspect of the spinal cord, at the level of C3-C4 (yellow arrow).  Note: intrathecal contrast is visualized along the ventral and dorsal aspects of the cord, without complete effacement of the CSF space. Furthermore, there are similar findings at C4-C5. (B) Axial view showing bilateral uncovertebral hypertrophy and facet arthrosis, greater on the right side compared to the left, contributing to severe right and moderate left foraminal narrowing.

In the hospital, his workup was concerning for myelopathy with upper motor neuron signs and a normal B12 level, and other lab findings were unremarkable. CT myelogram revealed severe spinal canal narrowing, superimposed on congenital spinal canal narrowing, worst at C3-C4 with signs of cord compression seen on both sagittal (Figure 1A) and axial views (Figure 1B). Neurosurgery was consulted, and the patient underwent a C3-C5 laminectomy. Rheumatology was consulted for polyarticular swelling and increased inflammatory markers and confirmed the patient had a pseudogout flare with arthrocentesis, which improved substantially with colchicine. Following these treatments, the patient began to improve considerably and returned to the IRF, where he received intensive therapy and was eventually discharged home. 

## Discussion

This case discusses the effects of a presumed diagnosis without definitive findings. In this specific case, the patient was believed to have SCD because of his presentation with multiple falls due to loss of balance and lab work showing anemia with low B12 levels and elevated MMA and homocysteine. He could not have an MRI due to retained bullet fragments and, therefore, was treated with B12 injections for presumed SCD. With this presentation, he was treated for an adequate period during the inpatient and rehabilitation stay, but his condition continued to progress. Usually, patients with SCD should improve significantly with B12 supplementation. In this case, electrodiagnostic studies may have assisted in the differential diagnosis, as SCD can show evidence of axonal degeneration, demyelination, or mixed features in nerve conduction studies [3,4]. With spinal stenosis, electrodiagnostic studies are not needed for diagnosis but can be helpful in those with myelopathic or radicular pain [5]. However, the most appropriate step for this patient was further imaging, as it would differentiate SCD from cervical myelopathy.

Given their similar presentations, SCD and cervical myelopathy are often on the differential diagnosis in patients with weakness and ataxia. However, in some, both are simultaneously present. Patel et al. in 2011 described the dual pathology of both spinal stenosis and vitamin B12 deficiency [6]. A retrospective study was performed where patients from an outpatient spine clinic were surveyed, and of the 457 patients included, 8.5% had vitamin B12 deficiency. Seventy-three percent of those underwent MRI/CT imaging, and 59% were noted to have spinal stenosis. Of those who were deficient, 64% were on treatment, and 72% endorsed improved outcomes on the supplements. The conclusion of this study was for spine specialists to be aware of the potential coexistence of B12 deficiency, especially in elderly patients with sensory disturbances, and that it may be worth screening prior to proceeding with spinal surgery. Our presented case argues that the opposite also holds value, in that if a patient with presumed SCD is not improving with treatment, that spinal stenosis could be contributing to their presentation. 

This case also shows the contrast between Occam’s razor and Hickam’s dictum. Occam’s razor describes where the simplest explanation is the likely solution, whereas Hickam’s dictum describes where “a patient can have as many diagnoses as he darn well pleases” [7]. Synthesizing a unified diagnosis, like Occam’s razor, for this patient with known B12 deficiency who had a dramatic functional decline with the setting of fever, disseminated joint pain, and elevated inflammatory markers was challenging. However, further workup at the acute hospital showed he had multiple, separate conditions that were acutely active with the worsening cervical changes and new findings of pseudogout like Hickam’s dictum.

## Conclusions

In patients with weakness, paresthesias, and ataxia, it is important to consider various etiologies that can contribute to this presentation. The differential diagnosis is broad and may include dual pathologies. This case illustrated a patient who had vitamin B12 deficiency thought to be SCD, which placed an anchoring bias when he presented with similar symptoms rather than attributing it to his cervical stenosis, which had progressed over time. It further supports the dichotomy between Occam’s razor and Hickam’s dictum and how it is important to note its influence on medical diagnostics. In this case, his condition appeared to worsen over the span of a week with his functional status and diffuse joint pain with evidence of inflammation, which puzzled many treating and consulting physicians in coming up with a unifying diagnosis when it was multiple conditions simultaneously occurring. Consequently, this case demonstrates the importance of formulating a broad differential diagnosis and also realizing when to question the diagnosis if a patient’s clinical course does not improve with treatment as expected.
